# Monoclonal Antibodies and Osteonecrosis of the Jaw: A Systematic Review of Risk, Incidence, and Clinical Management in Dentistry

**DOI:** 10.1155/ijod/2557594

**Published:** 2025-08-28

**Authors:** Gianna Dipalma, Alessio Danilo Inchingolo, Mariafrancesca Guglielmo, Irene Palumbo, Lilla Riccaldo, Roberta Morolla, Francesco Inchingolo, Daniela Di Venere, Andrea Palermo, Angelo Michele Inchingolo

**Affiliations:** ^1^Department of Interdisciplinary Medicine, University of Bari “Aldo Moro”, Bari 70121, Italy; ^2^Department of Biomedical, Surgical and Dental Sciences, Milan University, Milan 20122, Italy; ^3^Department of Experimental Medicine, University of Salento, Lecce 73100, Italy

**Keywords:** antibody therapy, BRONJ, denosumab, DRONJ, monoclonal antibodies, MRONJ, ONJ, osteonecrosis of the jaw

## Abstract

**Aim:** Osteonecrosis of the jaw (ONJ) is a complication associated with antiresorptive therapies, particularly bisphosphonates and monoclonal antibodies such as denosumab. This systematic review explores preventive measures, therapeutic strategies, and the role of a multidisciplinary approach in reducing the risk of ONJ.

**Materials and Methods:** The study was conducted following Preferred Reporting Items for Systematic Reviews and Meta-Analyses (PRISMA) guidelines, and the protocol was registered on PROSPERO (ID CRD42024593760). A literature search was performed across PubMed, Scopus, and Web of Science using the keywords “osteonecrosis of the jaw” OR “jaw osteonecrosis” OR “ONJ” AND (“monoclonal antibodies” OR “denosumab” OR “antibody therapy”). A total of 2629 articles were identified, of which 19 were included in the qualitative analysis.

**Results:** Denosumab provides significant benefits in treating bone-related conditions but carries a notable risk of ONJ, particularly in cancer patients and individuals undergoing invasive dental procedures.

**Conclusions:** Pretreatment dental assessments and personalized care are crucial for preventing ONJ. Surgical intervention has been shown to be the most effective treatment for resolving the condition.

## 1. Introduction

Osteonecrosis of the jaw (ONJ) is an uncommon yet significant complication associated with the use of antiresorptive therapies, particularly bisphosphonates and monoclonal antibodies such as denosumab [1–5].

Identified in 2003, ONJ presents as exposed necrotic bone in the maxillofacial region persisting for at least 8 weeks despite clinical intervention. Its diagnosis excludes other potential causes, such as craniofacial radiation or metastatic disease [6–11].

While rare, ONJ poses substantial challenges due to its implications for oral health, quality of life, and adherence to therapy, making it a critical concern in both dentistry and medicine [12–16].

Antiresorptive agents are essential in treating bone-related conditions like osteoporosis, metastatic bone disease, and malignancy-associated hypercalcemia. Bisphosphonates inhibit osteoclast-mediated bone resorption, stabilizing bone density and reducing fracture risk [17, 18].

Monoclonal antibodies, especially denosumab, have become pivotal in managing bone-related disorders due to their targeted mechanisms of action. Denosumab, a fully human monoclonal antibody, specifically inhibits the activity of receptor activator of nuclear factor-kappa B ligand (RANKL), a key mediator in osteoclast differentiation and activation. By suppressing osteoclast function, denosumab effectively reduces bone resorption, increases bone density, and decreases fracture risk in patients with osteoporosis and cancer-related bone conditions [16, 19]. However, its unique pharmacodynamics also contribute to the development of ONJ, particularly in high-dose regimens for oncology patients. Unlike bisphosphonates, which integrate into bone matrix and exhibit prolonged skeletal retention, denosumab has a reversible effect on bone turnover, making “drug holidays” a potential strategy to mitigate ONJ risks [16]. Despite this advantage, evidence indicates that denosumab-related ONJ (DRONJ) often presents more aggressively, with spontaneous onset or progression from early to advanced stages triggered by minor trauma or dental procedures. This underscores the need for rigorous preventive care, timely diagnosis, and multidisciplinary management to balance its therapeutic benefits with associated risks [20, 21]. The increasing use of denosumab in dental and oncological contexts highlights its dual potential to improve bone health while posing challenges for oral and systemic care, warranting ongoing investigation into optimized clinical guidelines [1, 19, 22–25].

First described in 2003, ONJ was initially reported in cancer patients treated with high-dose zoledronate or pamidronate for bone metastases. Over time, cases were also identified in osteoporosis patients, although at a much lower frequency [26–33].

Symptoms of ONJ include nonhealing bone lesions, pain, inflammation, and, in advanced stages, pathological fractures or fistulae [34, 35]. These symptoms underscore the importance of early diagnosis and intervention [36–44]. Diagnosis is primarily clinical, supported by patient history and physical examination.

The American Association of Oral and Maxillofacial Surgeons (AAOMS) has recently (2014) updated its definition of medication-related ONJ (MRONJ) to:

1. current or previous treatment with antiresorptive or antiangiogenic agents.
2. exposed bone or bone that can be probed through an intraoral or extraoral fistula (*e*) in the maxillofacial region that has persisted for more than 8 weeks.
3. no history of radiation therapy to the jaws or obvious metastatic disease to the jaws [45, 46].

ONJ is multifactorial, involving suppressed bone remodeling, infections, vascular impairment, and mucosal trauma [47, 48]. Bisphosphonates and denosumab, key players in ONJ, significantly reduce bone turnover [49, 50]. Bisphosphonates bind to bone hydroxyapatite and induce osteoclast apoptosis, while denosumab inhibits RANKL, suppressing osteoclast differentiation and activity [20, 21, 51–54].

Both mechanisms, while beneficial for bone conditions, can impair healing, particularly after invasive dental procedures [55–62].

Other contributing factors include bacterial infections that exacerbate local inflammation, systemic conditions like diabetes, and medications such as glucocorticoids, which impede wound healing and increase infection risk [58, 63–66]. The complex interplay of these factors underscores ONJ's multifaceted nature [67–70]. The incidence of ONJ varies by treatment and population. Among osteoporosis patients, the risk is exceptionally low, estimated at fewer than one case per 1000 patients annually [71–73]. Conversely, in cancer patients receiving high-dose antiresorptives for bone metastases, ONJ occurs in 1%–10% of cases, reflecting the dose-dependent nature of the condition [74–83].

Key risk factors include the cumulative dose and duration of antiresorptive therapy, invasive dental procedures (e.g., extractions), poor oral hygiene, and concurrent use of glucocorticoids or antiangiogenic agents. Additional risks include smoking, age, diabetes, and pre-existing periodontal disease [84–91]. Emerging evidence suggests genetic predispositions, such as specific polymorphisms in bone metabolism genes, may also influence ONJ susceptibility [92–98].

Managing ONJ requires a balance between the benefits of antiresorptive therapy and the risks of complications. For osteoporosis patients, the benefits of reduced fracture risk often outweigh the minimal ONJ risk. In oncology, where high-dose treatments are critical for managing skeletal complications, preventive strategies are vital [3, 99, 100].

Preventive measures focus on optimizing oral health before initiating therapy, avoiding invasive dental procedures during treatment, and maintaining stringent oral hygiene. For patients undergoing extractions or surgeries, temporary discontinuation of antiresorptive therapy (“drug holidays”) may be considered. Denosumab's reversible effects make it particularly suited for this approach, though the long skeletal retention of bisphosphonates limits immediate benefits from therapy discontinuation. The practice remains debated and should be tailored to individual risk profiles.

The objective of this systematic review is to critically examine the existing scientific literature on the use of monoclonal antibodies, particularly denosumab, in the management of osteoporosis and related conditions within the field of dentistry. This review aims to explore the incidence, risk factors, and clinical manifestations of MRONJ associated with monoclonal antibody therapies. This review seeks to identify preventive measures, therapeutic strategies, and the role of multidisciplinary approaches in mitigating the risks of MRONJ. The ultimate goal is to provide a comprehensive and evidence-based resource to support clinicians in optimizing dental and oncological care for patients undergoing monoclonal antibody treatment, while informing future research to refine clinical guidelines.

## 2. Materials and Methods

### 2.1. Protocol and Registration

The protocol was registered at PROSPERO with the ID CRD42024593760, and the systematic review was carried out following the Preferred Reporting Items for Systematic Reviews and Meta-Analyses (PRISMA) guidelines.

A meta-analysis was not performed due to the marked heterogeneity among the included studies in terms of study design, sample characteristics, types of interventions, outcome measures, and follow-up periods. This variability did not allow for a meaningful quantitative synthesis of the data.

### 2.2. Search Processing

Papers related to ONJs were found by monoclonal antibodies by searching PubMed, Scopus, and Web of Science between 1 January 2009 and 1 June 2024. (“osteonecrosis of the jaw” OR “jaw osteonecrosis” OR “ONJ”) AND (“monoclonal antibodies” OR “denosumab” OR “antibody therapy”) were the Boolean keywords utilized in the search strategy (Table 1).

### 2.3. Inclusion Criteria

The following were the inclusion criteria: (1) studies that examined the ONJs by monoclonal antibodies; (2) randomized clinical trials, retrospective studies, case-control studies, case series, case reports, and prospective studies; (3) English language; and (4) full-text studies.

Papers that did not match the above criteria were excluded.

The review was conducted using the following PICOS criteria:

Participants: Patients treated with monoclonal antibodies (e.g., denosumab) for osteoporosis, metastatic bone disease, or related conditions.

Interventions: Monoclonal antibody therapy, with preventive and therapeutic strategies for ONJ, including drug holidays and surgical or conservative treatments.

Comparisons: ONJ risk and outcomes with monoclonal antibodies versus other antiresorptive agents (e.g., bisphosphonates) and different management approaches.

Outcomes: ONJ incidence, risk factors, and effectiveness of prevention and treatment strategies.

Study: Randomized clinical trials, retrospective studies, case-control studies, case series, case reports, and prospective studies.

### 2.4. Exclusion Criteria

The exclusion criteria were as follows: (1) study involving animals; (2) irrelevant subjects; (3) reviews, letters, or comments; and (4) non-English language.

### 2.5. Data Processing

Three reviewers (Mariafrancesca Guglielmo, Irene Palumbo, and Roberta Morolla) independently searched the databases to find the studies and evaluated their quality based on the selection criteria. The selected articles were downloaded using version 6.0.15 (Corporation for Digital Scholarship, Vienna, VA, USA). To settle any disputes between the three authors, a senior reviewer (Francesco Inchingolo) was consulted.

### 2.6. Quality Assessment

The quality of the included papers was assessed by three reviewers, Mariafrancesca Guglielmo, Irene Palumbo, and Roberta Morolla, using the ROBINS tool [101].

Each of the seven points under evaluation had a bias level assigned to it. A third reviewer (Francesco Inchingolo) was consulted in cases of disagreement until an agreement was reached. Among the domains evaluated by ROBINS were the following:

• Confounding bias.
• Bias resulting from exposure measurement.
• Bias in a study's participant selection.
• Bias resulting from postexposure intervention.
• Bias resulting from missing data.
• Bias resulting from outcome measurement.
• Bias in the presentation of the results.

## 3. Results

### 3.1. Study Selection

A total of 2629 publications (Scopus *N* = 1394, PubMed *N* = 610, and Web of Science *N* = 625) were found using the electronic database search; no papers were found through the manual search.

**Figure 1:**
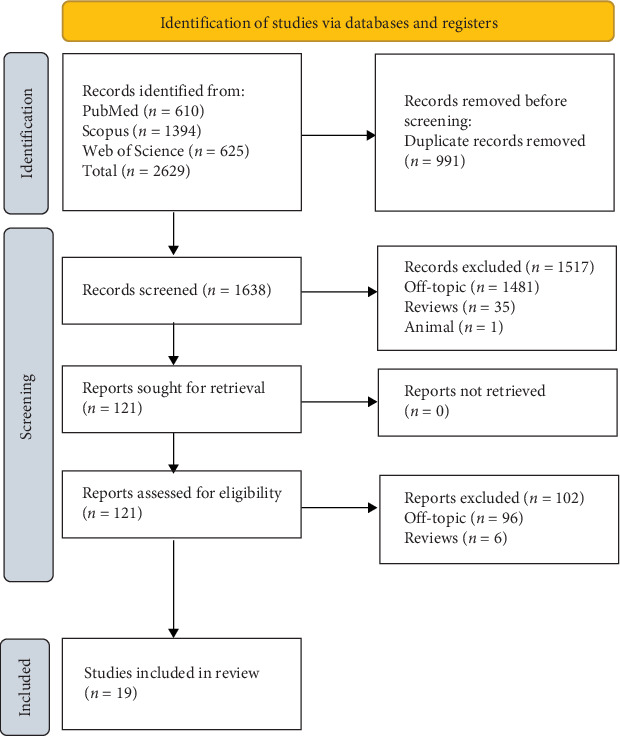
Literature search following the Preferred Reporting Items for Systematic Reviews and Meta-Analyses (PRISMA) flow diagram and database search indicators.

Following the removal of duplicates (*N* = 991), the abstracts and titles of 1638 studies were assessed for screening. Of these, 121 records were chosen for review after 1517 papers (1481 off-topic, 35 reviews, and 1 animal study) failed to meet the inclusion requirements. Of these, 102 reports (96 off-topic, 6 reviews) were eliminated because they did not fit the inclusion criteria. Nineteen records were chosen for a qualitative study after being deemed eligible.  Figure 1 depicts the selection procedure, and Table 2 provides a description of the records that were selected. In accordance with PRISMA guidelines, the PRISMA flowchart and PRISMA checklist have been included as Supporting Information. These documents provide a transparent overview of the study selection process and ensure adherence to standardized reporting criteria.

### 3.2. Methodological Features

The reviewed studies collectively illustrate the significant risk factors, incidence rates, and management strategies associated with DRONJ and MRONJ. Everts-Graber et al. [16] identified a higher incidence of ONJ in patients treated with denosumab compared to bisphosphonates, emphasizing the additional risk posed by sequential therapy. Jung et al. [111] noted that chronic inflammation and comorbidities, such as arthritis, are critical in predisposing patients to MRONJ. Voss et al. [70] highlighted the increased risk of ONJ recurrence among patients transitioning from bisphosphonates to denosumab, and Roato et al. [19] underscored immunological differences between denosumab and zoledronic acid as a potential determinant of disease severity and healing dynamics. Ikesue et al. and Fusco et al. [112, 113] demonstrated that denosumab is associated with a higher risk of MRONJ in cancer patients, particularly those undergoing invasive dental procedures or receiving antiangiogenic agents. Conversely, studies such as Liu et al. [117] showed a lower incidence of MRONJ in osteoporotic patients treated with denosumab, although long-term use remains a concern. Surgical approaches, as noted by Hoefert et al. [106] and Pichardo, consistently showed higher success rates in managing DRONJ, while conservative management yielded less favorable outcomes. Despite these findings, significant gaps persist in understanding the pathophysiology and optimal prevention strategies for DRONJ.

### 3.3. Quality Assessment and Risk of Bias of Included Articles

**Figure 2:**
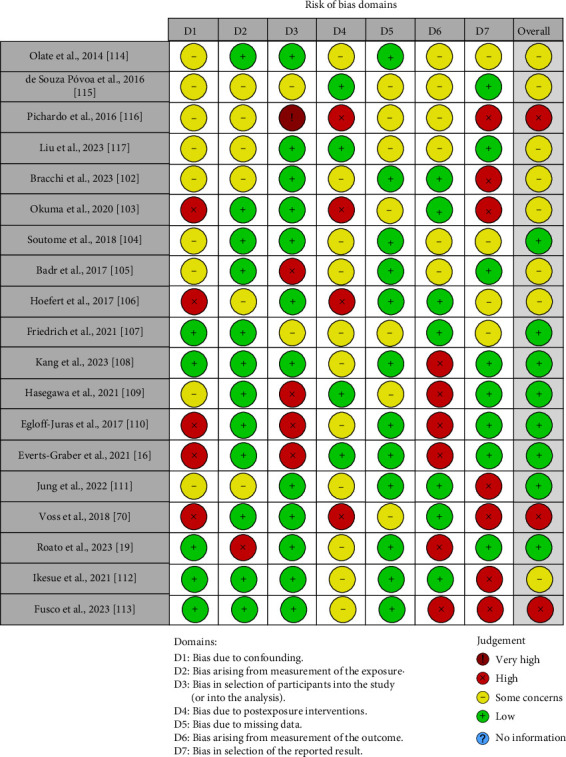
Bias assessment.

The risk of bias across the included studies was assessed using seven domains, as illustrated in the Figure 2. Overall, the quality of the studies varied significantly. Studies such as Liu et al. and Okuma et al. [103, 117] displayed predominantly low risks of bias across all domains, indicating robust methodologies. Conversely, Pichardo et al. and Hasegawa et al. [109, 116] showed notably high or very high risks of bias in domains D3 (selection of participants) and D6 (measurement of the outcome), raising concerns about their internal validity. Many studies, including Friedrich et al. and Kang et al. [107, 108], had high risks of bias due to missing data (D5) or the selection of reported results (D7). Furthermore, Egloff-Juras et al. and Roato et al. [19, 110] showed critical methodological limitations in D3 and D6. While some studies consistently demonstrated low bias in confounding (D1) and exposure measurement (D2), others, such as Voss et al. [70], had moderate concerns in these areas (Figure 2).

## 4. Discussion

Denosumab, a human monoclonal antibody targeting RANKL, is a potent antiresorptive agent increasingly used in metastatic cancer patients. Despite its efficacy, denosumab is associated with MRONJ, which poses challenges in clinical management [23].

### 4.1. MRONJ Risk and Comparison With Bisphosphonates

The risk of MRONJ associated with denosumab is a significant concern in oncology, with several studies indicating a higher incidence compared to bisphosphonates. For instance, Everts-Graber et al. and Ikesue et al. [16, 112] found that the incidence of MRONJ in denosumab-treated patients can reach up to 12.6%, while bisphosphonates like zoledronic acid are linked to a lower incidence of 4.4. This discrepancy can be attributed to the different mechanisms of action of these drugs. Bisphosphonates, which accumulate in the bone matrix, inhibit osteoclasts over an extended period, whereas denosumab, a monoclonal antibody, reversibly inhibits the RANKL, leading to a more pronounced but temporary suppression of bone resorption during active treatment [114]. Comorbidities, such as arthritis, diabetes, and chronic inflammation, were also found to increase susceptibility to DRONJ, as noted by Jung et al. [111] and Okuma et al. [103]. Furthermore, Fusco et al. [113] demonstrated that invasive dental procedures, alongside the concurrent use of antiangiogenic drugs, amplify the risk in oncology patients. The transient nature of denosumab's effect on bone turnover may predispose the bone to greater fragility during therapy, thereby increasing susceptibility to trauma and infection, which are key triggers for osteonecrosis [102].

### 4.2. Sequential Therapies and Risk Factors

In addition to the direct risks associated with denosumab, sequential therapies, particularly switching from bisphosphonates to denosumab, pose an additional challenge. As highlighted by Voss et al. [70], this sequential therapeutic strategy may result in cumulative adverse effects on bone metabolism, amplifying the risk of osteonecrosis and increasing the likelihood of recurrence. Therefore, transitioning from bisphosphonates to denosumab should be carefully considered, with possible implementation of a “drug holiday” to mitigate cumulative risks.

Furthermore, other well-established risk factors for MRONJ, including advanced age (particularly over 65 years), prior dental extractions, and comorbidities such as rheumatoid arthritis and diabetes, also contribute significantly to the onset of MRONJ [110]. These factors were evident in studies by Jung et al. and Ikesue et al. [111, 112], who underscored the importance of thorough pretreatment dental assessments and individualized management plans to mitigate these risks. The high incidence of MRONJ in patients who have undergone tooth extractions, particularly in those receiving denosumab, highlights the need for strict dental monitoring during therapy, as demonstrated in the studies by Egloff-Juras et al. and Hasegawa et al. [109, 110].

### 4.3. Prevention and Management Strategies

The importance of preventive dental care is underscored by Soutome et al. [104], who demonstrated that pre-existing dental infections are a stronger predictor of ONJ than surgical extractions. Early dental evaluations and regular follow-ups are pivotal in reducing ONJ incidence, as highlighted by Okuma et al. [103]. Management strategies favor surgical interventions, as evidenced by Hoefert et al. and Pichardo [106, 116], who reported higher success rates with surgical debridement compared to conservative methods. Kang et al. [108] further corroborated these findings, although recurrences remain a concern in some cases. To provide a clearer and more accessible overview, Table 3 summarizes the key prevention and management strategies for DRONJ identified across the 19 included studies.

### 4.4. Immunological Differences and Healing Outcomes

An interesting immunological distinction between denosumab and bisphosphonates was revealed by Roato et al. [19], which may explain the differences in healing outcomes between the two treatments. Denosumab appears to induce less immunosuppression than bisphosphonates, with patients on denosumab exhibiting higher levels of activated T cells and γδ T cells, potentially leading to a more robust immune response to tissue damage. As a result, MRONJ cases associated with denosumab tend to show faster resolution after discontinuation, as evidenced in studies by Ikesue et al. [112]. The reversibility of denosumab's effects on bone turnover offers a potential therapeutic advantage, allowing for quicker healing of necrotic lesions compared to those induced by bisphosphonates. de Souza Póvoa and others delved into denosumab's unique mechanism of action, blocking the RANK–RANKL signaling pathway extracellularly, leading to reversible bone turnover inhibition, a feature that underpins its distinct clinical outcomes [115].

This consideration may influence clinicians in decisions regarding the management and treatment of DRONJ.

### 4.5. Cancer-Specific Considerations

In oncology, Fusco et al. and Badr et al. pointed out the aggressive nature of DRONJ, particularly in patients receiving high doses of denosumab [105, 113]. Antiangiogenic drugs and prolonged treatment duration exacerbate risks, while minor traumas or dental interventions often act as triggers. Despite these challenges, studies like Liu et al. [117] suggest a lower incidence of ONJ in osteoporotic patients, indicating a differential risk profile based on underlying conditions and treatment regimens.

Moreover, a case report describes a rare phenomenon of mandibular reossification during denosumab therapy in a 44-year-old patient with metastatic breast cancer. Initially, the patient presented with a sharply defined osteolytic lesion in the mandibular ramus, identified as a metastasis. Denosumab was initiated alongside chemotherapy and hormone therapy. Seven months later, imaging revealed significant calcification of the defect, with ossification resembling callus formation, although new metastases had developed elsewhere. The case highlights denosumab's potential to reduce tumor-associated mandibular osteolysis while preventing further bone degradation. This finding underscores the importance of multidisciplinary evaluation in interpreting mandibular lesions in oncology patients [107].

Kang et al. examined four cases of DRONJ among 74 cancer patients treated with high-dose denosumab (120 mg/month) for metastatic cancer. Three patients had prostate cancer, and one had breast cancer. Tooth extraction within 2 months of the last denosumab injection was identified as a risk factor in three cases, while the fourth occurred spontaneously. Pathological findings revealed acute and chronic inflammation, including *Actinomyces* colonies. Three patients underwent successful surgical treatment without complications or recurrence; one experienced DRONJ recurrence at a different site, which resolved with sequestrectomy, antibiotics, and denosumab discontinuation. Healing occurred within an average follow-up of 5 months. These findings underscore the importance of surgical intervention combined with conservative medical management in treating DRONJ [108].

### 4.6. Impact of Antiangiogenic Therapies

The combination of denosumab with antiangiogenic therapies, such as bevacizumab and sunitinib, has been shown to further exacerbate the risk of MRONJ. Fusco et al. [113] suggest that antiangiogenic agents may impair bone vascularization, which in turn reduces the tissue's capacity to heal, thereby increasing the likelihood of necrosis. Additionally, the prolonged survival of cancer patients due to advances in modern oncology treatments, coupled with the extended exposure to agents like denosumab, raises the cumulative risk of MRONJ. These findings highlight the need for vigilant monitoring and multidisciplinary approaches to manage MRONJ, particularly in cancer patients receiving long-term therapy with denosumab and antiangiogenic agents.

### 4.7. Surgical and Conservative Management of DRONJ

As noted by several studies, including Hasegawa et al. and Pichardo et al., treatment approaches for DRONJ range from conservative management to more invasive surgical interventions [109, 116]. Surgical debridement has shown higher success rates, especially in severe cases of necrosis. Hasegawa et al. found that major surgical interventions led to an 80% healing rate, significantly outperforming nonsurgical treatments, which had only a 20% success rate. Conversely, the effectiveness of a “drug holiday” or temporary discontinuation of denosumab in the management of DRONJ remains debatable. Studies by Egloff-Juras et al. [110] and others have suggested that while drug holidays may not significantly impact healing in all cases, they could play a role in reducing the progression of osteonecrosis, particularly when combined with surgical debridement [115].

### 4.8. Prevention and Future Research

Despite these advances, the optimal prevention and management strategies for DRONJ remain under investigation. Several studies emphasize the importance of preventive dental care, including pretreatment dental evaluations and regular follow-up during denosumab therapy. As noted by Bracchi et al. and Okuma et al., effective prevention hinges on managing pre-existing dental conditions, such as periodontitis or apical lesions, which are significant risk factors for the development of MRONJ. These findings call for a standardized, multidisciplinary approach to patient management that includes oncologists, dentists, and oral surgeons.

## 5. Limitations

The limitations of this review concern several aspects. First, the variability of clinical data presents a significant challenge, as studies are based on heterogeneous patient populations with differences in comorbidities, treatment protocols, and concomitant medications, factors that may influence outcomes related to the risk of osteonecrosis. Furthermore, most research focuses on short- and medium-term effects, with a lack of long-term data on the risk of osteonecrosis in patients treated with denosumab. Diagnostic inconsistencies also present a problem, as variations in diagnostic criteria and methodologies between studies limit the ability to compare outcomes, particularly with regard to the diagnosis of MRONJ. Furthermore, the lack of a standardized approach to the prevention of MRONJ, coupled with variations in clinical practice, makes it difficult to assess the effectiveness of preventive measures. Finally, the limited number of studies on the interaction between denosumab and other drugs, such as antiangiogenics, hinders a comprehensive understanding of their combined impact on bone health.

## 6. Conclusions

In conclusion, while denosumab presents a lower risk of MRONJ compared to bisphosphonates, its impact on bone metabolism and the potential for osteonecrosis, especially in cancer patients receiving prolonged therapy, necessitate careful management. Key risk factors such as dental treatments, age, and comorbidities must be closely monitored, and a more comprehensive understanding of the underlying mechanisms of DRONJ is necessary to refine preventive and therapeutic strategies. Further research into the interactions between denosumab, antiangiogenic agents, and other treatments, as well as long-term outcomes, is crucial to improving patient care and minimizing the risks of MRONJ.

## Figures and Tables

**Table 1:** Full search strings for each database.

PubMed	(“osteonecrosis of the jaw” OR “jaw osteonecrosis” OR “ONJ”) AND (“monoclonal antibodies” OR “denosumab” OR “antibody therapy”)

Scopus	(“osteonecrosis of the jaw” OR “jaw osteonecrosis” OR “ONJ”) AND (“monoclonal antibodies” OR “denosumab” OR “antibody therapy”)

Web of Science	(“osteonecrosis of the jaw” OR “jaw osteonecrosis” OR “ONJ”) AND (“monoclonal antibodies” OR “denosumab” OR “antibody therapy”)

**Table 2:** Descriptive summary of item selection.

Author (year)	Study design	Number of patients	Average age and gender	Monoclonal antibody used	Outcomes
Bracchi et al. (2023) [102]	Retrospective observational study	780	65 years	Denosumab	Nine patients reported full MRONJ resolution by the end of the follow-up, 23 were still undergoing treatment or lost to follow-up.

Okuma et al. (2020) [103]	Retrospective single-center observational study	123	55–68 years, 57 males and 66 females	Denosumab	The occurrence and progression of DRONJ were assessed, with some patients experiencing stabilization or aggressive progression.

Soutome et al. (2018) [104]	Retrospective multicenter study	135	62.6 years, 64 were males (47.4%) and 71 were females (52.6%)	Bisphosphonates (BPs): 80 patients and denosumab (Dmab): 55 patients	18 out of 135 patients (13.3%) developed MRONJ. The 1-year, 2-year, and 3-year cumulative occurrence rates were 8.6%, 21.5%, and 29.2%, respectively.

Badr et al. (2017) [105]	Case series	4	55–76 years, all females	Denosumab	Aggressive MRONJ progression, stages 0–3. Some improvement was noted after denosumab discontinuation (drug holiday).

Hoefert et al. (2017) [106]	Retrospective cohort study	17	68.5 years, 10 females and 7 males	Denosumab	DRONJ occurred after 14 doses; major surgery achieved 80% healing, nonoperative 20%; cessation had no effect.

Friedrich et al. (2021) [107]	Case report	1	44 years, female	Denosumab	Significant reossification of a mandibular metastasis after 7 months of denosumab therapy, despite new metastases elsewhere.

Kang et al. (2023) [108]	Retrospective case series	4	75 years, 3 males and 1 female	Denosumab	DRONJ linked to tooth extractions; surgical treatment, antibiotics, and denosumab cessation achieved healing in 5 months.

Hasegawa et al. (2021) [109]	Multicenter retrospective study	72	65.2 years, 41 women and 31 men	Denosumab	DRONJ incidence: 28.7%; risk factors include inflammation, corticosteroids, periodontitis, and extended treatment duration.

Egloff-Juras et al. (2017) [110]	Retrospective cohort study	141	58.9 years, mixed gender	Denosumab	DRONJ incidence: 6.4%; dental extractions significantly increased risk; associated with injuries or spontaneously.

Everts-Graber et al. (2021)[16]	Retrospective cohort	9956	72 years, male/female	Denosumab	Higher risk of MRONJ with denosumab compared to bisphosphonates (28.3 vs. 4.5 per 10,000 person-years).

Jung et al. (2022) [111]	Retrospective cohort	98	70.5 years, male/female	Denosumab	MRONJ incidence of 4.1%. Higher risk associated with periodontal inflammation and arthritis.

Voss et al. (2018) [70]	Retrospective cohort	52	70 years, male/female	Denosumab	Higher recurrence rate of MRONJ when switching from bisphosphonates to denosumab (45.5% vs. 17.6%).

Roato et al. (2023) [19]	Retrospective cohort	10	Oncology patients with metastases	Denosumab	Denosumab is less immunosuppressive than zoledronic acid; better MRONJ healing outcomes.

Ikesue et al. (2021)[112]	Retrospective cohort	374	68–69 years, male/female	Denosumab and zoledronic acid	MRONJ incidence: denosumab (12.6%) > zoledronic acid (4.4%). Faster resolution with denosumab (26.8 months).

Fusco et al. (2023) [113]	Retrospective cohort	335	Predominantly female (72%)	Denosumab and bevacizumab	Increased MRONJ cases linked to denosumab and antiangiogenic drugs.

Olate et al. (2014) [114]	Case report	1	55 years, female	Denosumab	No significant changes in the clinical presentation 14 months after the dental extraction.

de Souza Póvoa et al. (2016) [115]	Case report	1	58 years, male	Denosumab	Conservative treatment was successful.

Pichardo et al. (2016) [116]	Retrospective case series	11	72.6 years, 7 females and 4 males	Denosumab	Healing of DRONJ after surgical treatment.

Liu et al. (2023) [117]	Multicenter study	8962	Not available	Denosumab	The incidence was 2.49 per 1000 patient-years in the bisphosphonate (BP) group and 1.47 per 1000 patient-years in the denosumab group.

**Table 3:** Prevention and management strategies in DRONJ.

Category	Strategy	Number of studies reporting it	Representative references
Prevention	Pretreatment dental evaluation and oral hygiene optimization	6/19	Okuma et al. [103], Soutome et al. [104], Jung et al. [111], Hasegawa et al. [109], Bracchi et al. [102], Fusco et al. [113]
	Treatment of periodontal disease or infections before therapy	4/19	Soutome et al. [104], Jung et al. [111], Hasegawa et al. [109], Bracchi et al. [102]
	Avoidance or careful planning of dental extractions	5/19	Egloff-Juras et al. [110], Kang et al. [108], Okuma et al. [103], Hasegawa et al. [109], Fusco et al. [113]
	Avoidance of antiangiogenic agents in combination with denosumab	2/19	Fusco et al. [113], Roato et al. [19]
	Risk factor assessment (age, comorbidities, drug history)	4/19	Jung et al. [111], Ikesue et al. [112], Voss et al. [70], Soutome et al. [104]

Management	Surgical treatment (debridement, sequestrectomy, resection)	9/19	Hoefert et al. [106], Kang et al. [108], Pichardo et al. [116], Hasegawa et al. [109], Okuma et al. [103], de Souza Póvoa et al. [115], Roato et al. [19], Voss et al. [70], Badr et al. [105]
	Conservative treatment (antibiotics, mouth rinses, follow-up)	6/19	Bracchi et al. [102], Hasegawa et al. [109], de Souza Póvoa et al. [115], Okuma et al. [103], Pichardo et al. [116], Badr et al. [105]
	Drug holiday (temporary discontinuation of denosumab)	4/19	Badr et al. [105], Kang et al. [108], Ikesue et al. [112], Egloff-Juras et al. [110]
	Combined surgical and conservative approach	3/19	Kang et al. [108], Pichardo et al. [116], Hasegawa et al. [109]
	Spontaneous resolution/observation only	2/19	Bracchi et al. [102], Friedrich et al. [107]

## Data Availability

No new data were created or analyzed in this study. The data sharing is not applicable to this article.

## Nomenclature

DRONJ: Denosumab-related osteonecrosis of the jaw
MRONJ: Medication-related osteonecrosis of the jaw
ONJ: Osteonecrosis of the jaw
RANKL: Receptor activator of nuclear factor-kappa B ligand.

## Supporting Information

Supporting Information The PRISMA 2020 checklist for the present systematic review is provided as a supporting file. This checklist documents adherence to the PRISMA 2020 guidelines, ensuring transparent and comprehensive reporting of the review process.
